# malERA: An updated research agenda for malaria elimination and eradication

**DOI:** 10.1371/journal.pmed.1002456

**Published:** 2017-11-30

**Authors:** Regina N. Rabinovich, Chris Drakeley, Abdoulaye A. Djimde, B. Fenton Hall, Simon I. Hay, Janet Hemingway, David C. Kaslow, Abdisalan Noor, Fredros Okumu, Richard Steketee, Marcel Tanner, Timothy N. C. Wells, Maxine A. Whittaker, Elizabeth A. Winzeler, Dyann F. Wirth, Kate Whitfield, Pedro L. Alonso

**Affiliations:** 1 ISGlobal, Barcelona Ctr. Int. Health Res. (CRESIB), Hospital Clínic—Universitat de Barcelona, Barcelona, Spain; 2 Harvard T.H. Chan School of Public Health, Boston, Massachusetts, United States of America; 3 London School of Hygiene and Tropical Medicine, London, United Kingdom; 4 University of Science, Techniques and Technology of Bamako, Bamako, Mali; 5 National Institute of Allergy and Infectious Diseases (NIAID) at the National Institutes of Health (NIH), Bethesda, Maryland, United States of America; 6 Institute for Health Metrics and Evaluation, University of Washington, Seattle, Washington, United States of America; 7 Big Data Institute, Li Ka Shing Centre for Health Information and Discovery, University of Oxford, Oxford, United Kingdom; 8 Liverpool School of Tropical Medicine, Liverpool, United Kingdom; 9 PATH Essential Medicines and PATH Center for Vaccine Innovation and Access, Seattle, Washington, United States of America; 10 KEMRI Wellcome Trust Research Programme, Nairobi, Kenya; 11 Centre for Tropical Medicine and Global Health, Nuffield Department of Clinical Medicine, University of Oxford, Oxford, United Kingdom; 12 Ifakara Health Institute, Ifakara, Tanzania; 13 School of Public Health, Faculty of Health Sciences, University of the Witwatersrand, Parktown, South Africa; 14 Institute of Biodiversity, Animal Health and Comparative Medicine, University of Glasgow, Glasgow, United Kingdom; 15 PATH Malaria Control and Elimination Partnership in Africa, Seattle, Washington, United States of America; 16 Swiss TPH, Basel, Switzerland; 17 University of Basel, Basel, Switzerland; 18 Medicines for Malaria Venture, Geneva, Switzerland; 19 College of Public Health, Medical and Veterinary Sciences, James Cook University, Townsville, Australia; 20 University of California, San Diego, School of Medicine, La Jolla, California, United States of America; 21 World Health Organization (WHO), Global Malaria Programme (GMP), Geneva, Switzerland

## Abstract

Achieving a malaria-free world presents exciting scientific challenges as well as overwhelming health, equity, and economic benefits. WHO and countries are setting ambitious goals for reducing the burden and eliminating malaria through the “Global Technical Strategy” and 21 countries are aiming to eliminate malaria by 2020. The commitment to achieve these targets should be celebrated. However, the need for innovation to achieve these goals, sustain elimination, and free the world of malaria is greater than ever. Over 180 experts across multiple disciplines are engaged in the Malaria Eradication Research Agenda (malERA) Refresh process to address problems that need to be solved. The result is a research and development agenda to accelerate malaria elimination and, in the longer term, transform the malaria community’s ability to eradicate it globally.

## Abstract 2

The malERA Refresh Consultative Panel on Health Systems and Policy Research summarize a research and development agenda to accelerate malaria elimination and eradicate globally.

**Summary points**

- The first malERA consultative process in 2011 identified a number of targets for investment and the scientific community has made progress across the research and development (R&D) continuum. Progress includes positive scientific opinion for a malaria vaccine, advanced development of 3 nonpyrethroid insecticides, new genetic technologies with the potential to alter malaria parasite transmission by the mosquito, identification of markers of drug resistance, and development of *Plasmodium vivax* liver stage assays as well as new collaborative approaches to mathematical modelling and screening for active ingredients for drugs and insecticides.
- Scientific progress, however, has been matched with significant challenges. The expansion of both insecticide and drug resistance threatens progress in affected countries. Gaps in the knowledge base persist, from epidemiological and entomological tools to guide programmes, particularly at low transmission levels, understanding the role of low-density infections in maintaining transmission and developing appropriate diagnostics for programmes, biomarkers, and tools to detect and clear hypnozoites, to tools to tackle residual transmission, receptivity, and prevention of reintroduction.
- In some areas, progress has been too slow, particularly in the creation of a tool kit to tackle *P*. *vivax* malaria, investments in the development of new vector control tools, almost all aspects of entomology, and in systematically testing solutions in the context of the respective health and social systems.
- Malaria parasites and their infections continually evolve, creating new research and programme challenges. In one region, human infections with *P*. *knowlesi* are rising, parasites with hrp2/3 deletions are evading detection by current rapid diagnostic tests (RDTs), and current effective vector control tools are selecting for mosquitoes with both physiologic resistance and behavioural traits like outdoor biting and resting.
- The malERA Refresh agenda proposes a broad agenda for transdisciplinary solutions to the problems faced. It points to 3 areas in which innovation is critical: (i) iterative improvements in drugs and vector control; (ii) transformative improvements in tools and strategies to reduce, if not halt, the parasite’s capacity to transmit; and (iii) integrated approaches in which a robust elimination strategy responds to local variations in transmission dynamics, is tailored to the health and social system context, and draws strength from other sectors.

## Introduction

The 2011 malaria Eradication Research Agenda (malERA) was the first comprehensive analysis of the science needed to support national elimination of malaria and the long-term goal of its global eradication [1]. The 2011 malERA consultative process engaged a multidisciplinary group, involving members of the infectious disease and malaria research and implementation communities, and identified both emerging challenges and approaches to solving them. Five years later, the review of progress and emerging challenges, as well as a more nuanced understanding of the implementation problems that need to be solved, drove the 2016 ‘malERA Refresh’, with the intent to assess progress and the emergence of new challenges, examine current hypotheses, and point to the key research and development areas that can advance the feasibility of malaria elimination in the most challenging areas of the world.

Global goals for a reduction in malaria burden and elimination were published in 2 complementary documents in 2015: the Global Technical Strategy for Malaria 2016–2030 (GTS) and Action and Investment to defeat Malaria 2016–2030 (AIM), a global investment case for financing and coordinating these efforts [2,3]. Other groups have expressed a vision of global malaria eradication and underscored the need for R&D investments and country financing [4]. Building on the goals expressed in the GTS and AIM, the World Health Organization (WHO) has established a Strategic Advisory Group (SAG) to analyse future scenarios for malaria, including eradication. WHO SAG has affirmed WHO’s long-standing commitment to the goal of eradication, although it does not specify an end date for that goal [5,6].

There is not an assumption that 1 single ‘silver bullet’ will solve all of the challenges, but—as was stated by Tachi Yamada in 2007—’imperfect tools applied imperfectly can still achieve remarkable impact’, and a toolbox of solutions is needed that countries can draw upon and adapt to their health and social systems context [7,8]. A strong research base is a keystone for long-term progress in achieving the goals of the GTS. It is in this context that the malERA Refresh Panels propose a multidisciplinary research agenda for researchers, programme implementers, and research funders to accelerate problem solving and impact.

### Accelerating to elimination

**Fig 1:**
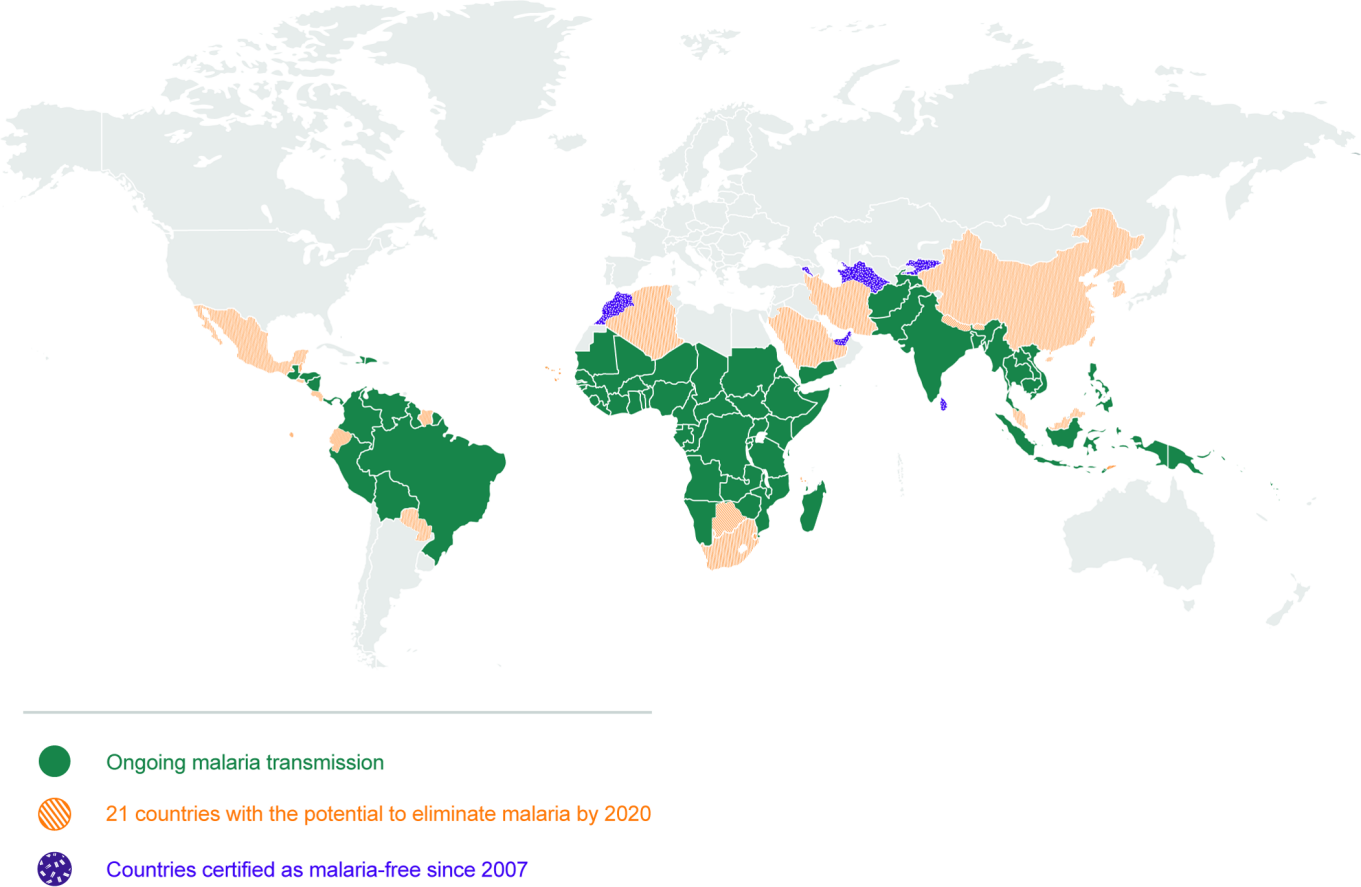
Map of 21 countries with the potential to eliminate malaria by 2020. There are 91 countries and territories with ongoing malaria transmission [9]. An analysis by WHO has identified 21 countries with the potential to eliminate by 2020: Algeria, Belize, Bhutan, Botswana, Cabo Verde, China, Comoros, Costa Rica, Ecuador, El Salvador, Iran (Islamic Republic of), Malaysia, Mexico, Nepal, Paraguay, Republic of Korea, Saudi Arabia, South Africa, Suriname, Swaziland, and Timor-Leste [10]. Countries and territories that have been certified malaria-free since 2007 are the United Arab Emirates (2007), Morocco (2010), Turkmenistan (2010), Armenia (2011), Maldives (2015), Sri Lanka (2016), and Kyrgyzstan (2016) [9,10]. Argentina and Paraguay have formally requested certification of malaria elimination and are in the process. Note that not all countries that have achieved zero indigenous cases for 3 consecutive years have sought certification from WHO. *Map base vector created by Freepik*.

Elimination of malaria means the ‘interruption of local transmission (reduction to zero incidence of indigenous cases) of a specified malaria parasite in a defined geographical area as a result of deliberate activities. Continued measures to prevent re-establishment of transmission are required’ (see Glossary, Table 1). A number of countries have been able or are on their way to eliminating malaria by applying a combination of vector control, efficient case management, and active surveillance strategies, all with existing tools for prevention, diagnosis, and treatment. Between 2000 and 2015, 17 countries eliminated malaria [9]. A further 21 countries have been identified as having the potential to eliminate malaria by 2020, comprising the “E-2020” (Fig 1) [10,11]. There are key elements to the elimination strategy, reflected in high uptake of core interventions by programmes and communities: a robust surveillance, reporting, and response system; prevention with a variety of ways to deliver insecticides and barrier methods to stop infectious bites; and diagnosis and treatment with effective combination medications. For this reason, WHO now frames national elimination as a continuum rather than the achievement of milestones for specific phases [6]. The heterogeneous nature of malaria across geographies means that a single approach will not work in all settings with the same efficiency. According to the ‘Acceleration Hypothesis’, countries with high vectorial capacity, particularly in sub-Saharan Africa, may require measures to rapidly deplete the parasite population [6,12], after which, locally tailored vector control, case management, and surveillance strategies with active methods to investigate and clear infections can then more effectively reduce transmission [12]. Whilst currently being considered and tested, strategies to accelerate elimination (such as mass drug administration [MDA] with antimalarials, low dose primaquine, complementary tools to address residual transmission, etc.) have not yet, and may not be, proven to be widely effective in moving settings with high residual transmission towards sustainable elimination. Ongoing research testing these tools and strategies is curated in the open MESA Track database [13]. Across the malaria endemic world, there exist challenges, and it is here that innovation is required to achieve elimination and quicken its course. Those challenges include areas of high receptivity (where the ecosystems are favourable for malaria transmission), highly competent vectors, residual transmission, resistance to drugs and/or insecticides, and areas where there are human populations that are not adequately served by the health system.

**Table 1:** Glossary of terms. The meaning of the terms used in the malERA Refresh series are described here. The sources of the definitions are referenced; where no reference is cited, the authors of this paper provided the definition.

Term	Definition	Reference
Asymptomatic parasitaemia	The presence of asexual parasites in the blood without symptoms of illness.	[14]
CHMI, also called human blood-stage challenge model	An established malaria infection model in which a group of healthy volunteers are inoculated with *Plasmodium* sporozoites via the bite of laboratory-reared infected female *Anopheline* mosquitoes or via needle and syringe, followed by complete medical cure. Volunteers are closely monitored for safety and clinical trial end points. CHMI allows the assessment of malaria vaccines, drugs, diagnostics, and the study of immunological mechanisms.	[15–17]
CRISPR	Gene-editing technology allowing for highly specific DNA modification. The technique is based on a bacterially derived endonuclease, such as Cas9, which can cut DNA in any desired location given a synthetic RNA guide sequence, the CRISPR. A new DNA sequence can then be introduced in that position by DNA repair machinery.	
Dormancy	Any state of suppressed development (developmental arrest) that is adaptive (that is, ecologically or evolutionarily meaningful and not just artificially induced) and usually accompanied by metabolic suppression (can apply to the parasite or vector).	[18]
Efficacy	A measure of the beneficial effect of an intervention in a controlled setting, for example, a randomised controlled trial.	
Effectiveness	A measure of to what extent the efficacy of an intervention can be retained at the individual (clinical) or the community (systems) level.	[19]
Elimination (of malaria)	Interruption of local transmission (reduction to zero incidence of indigenous cases) of a specified malaria parasite in a defined geographical area as a result of deliberate activities. Continued measures to prevent reestablishment of transmission are required. Note that the certification of malaria elimination in a country will require that local transmission is interrupted for all human malaria parasites.	[14]
Eradication (of malaria)	Permanent reduction to zero of the worldwide incidence of infection caused by human malaria parasites as a result of deliberate activities. Interventions are no longer required once eradication has been achieved.	[14]
Operational research	Any research producing practically usable knowledge (evidence, findings, information, etc.) that can improve programme implementation regardless of the type of research (design, methodology, approach).	[20]
Persistence	The continued presence of malaria parasites (in humans or mosquitoes) for an extended period, generally after initial intervention has concluded.	
Receptivity	Receptivity of an ecosystem to transmission of malaria. Note that a receptive ecosystem should have, e.g., the presence of competent vectors, a suitable climate, and a susceptible population.	[14]
Recrudescence	Recurrence of asexual parasitaemia of the same genotype(s) that caused the original illness, due to incomplete clearance of asexual parasites after antimalarial treatment. Note that recrudescence is different than reinfection with a parasite of the same or different genotype(s) and relapse in *P*. *vivax* and *P*. *ovale* infections.	[14]
Reinfection	A new infection that follows a primary infection; it can be distinguished from recrudescence by the parasite genotype, which is often (but not always) different than the genotype that caused the initial infection.	[14]
Relapse	Recurrence of asexual parasitaemia in *P*. *vivax* or *P*. *ovale* infections arising from hypnozoites. Note that relapse occurs when the blood-stage infection has been eliminated but hypnozoites persist in the liver and mature to form hepatic schizonts. After an interval, generally from 3 weeks to 1 year, the hepatic schizonts rupture and liberate merozoites into the bloodstream.	[14]
Residual transmission	Persistence of transmission after good coverage has been achieved with high-quality vector control interventions, to which local vectors are fully susceptible. Note that both human and vector behaviour is responsible for such residual transmission, such as people staying outdoors at night or local mosquito vector species displaying behaviour that allows them to avoid core interventions.	[14]
SERCaP	A description of an ideal antimalarial drug therapy, which, in a single-patient encounter, both eliminates all parasites in the patient and provides individual protection from reinfection for at least 1 month after treatment.	[21]
Malaria stratification	Classification of geographical areas or localities according to epidemiological, ecological, social, and economic determinants for the purpose of guiding malaria interventions.	[14]
Subpatent infection	Low-density blood-stage malaria infection that is not detected by standard diagnostic tools.	
Submicroscopic infection	Low-density blood-stage malaria infection that is not detected by conventional microscopy.	[14]
Surveillance	Continuous, systematic collection, analysis, and interpretation of disease-specific data and use in planning, implementing, and evaluating public health practice. Note that surveillance can be done at different levels of the healthcare system (e.g., health facilities, the community) with different detection systems (e.g., case-based: active or passive) and sampling strategies (e.g., sentinel sites, surveys).	[14]
Vector competence	For malaria, the ability of the mosquito to support completion of malaria parasite development after zygote formation and oocyst formation and development and release of sporozoites that migrate to salivary glands, allowing transmission of viable sporozoites when the infective female mosquito feeds again. Note that human malarias are transmitted exclusively by competent species of *Anopheles* mosquitoes; various plasmodia are transmitted by competent species of mosquitoes of the genera *Aedes*, *Anopheles*, and *Culex* and other haematophagous *Diptera*.	[14]
VIMT	Vaccines that target the sexual- and mosquito-stage antigens, pre-erythrocytic vaccines that reduce asexual- and sexual-stage parasite prevalence rates, asexual erythrocytic-stage vaccines that inhibit multiplication of asexual stage parasites, or vaccines that target vector antigens to disrupt parasitic development in the mosquito.	[22]
Vulnerability	The frequency of influx of infected individuals or groups and/or infective anopheline mosquitoes. Note that vulnerability is also referred to as ‘importation risk’. The term can also be applied to the introduction of drug resistance in a specific area.	[14]

**Abbreviations:** CHMI, controlled human malaria infection; CRISPR, clustered regularly interspaced short palindromic repeats; malERA, Malaria Eradication Research Agenda; SERCaP, Single-Encounter Radical Cure and Prophylaxis; VIMT, vaccines that interrupt malaria parasite transmission.

**Fig 2:**
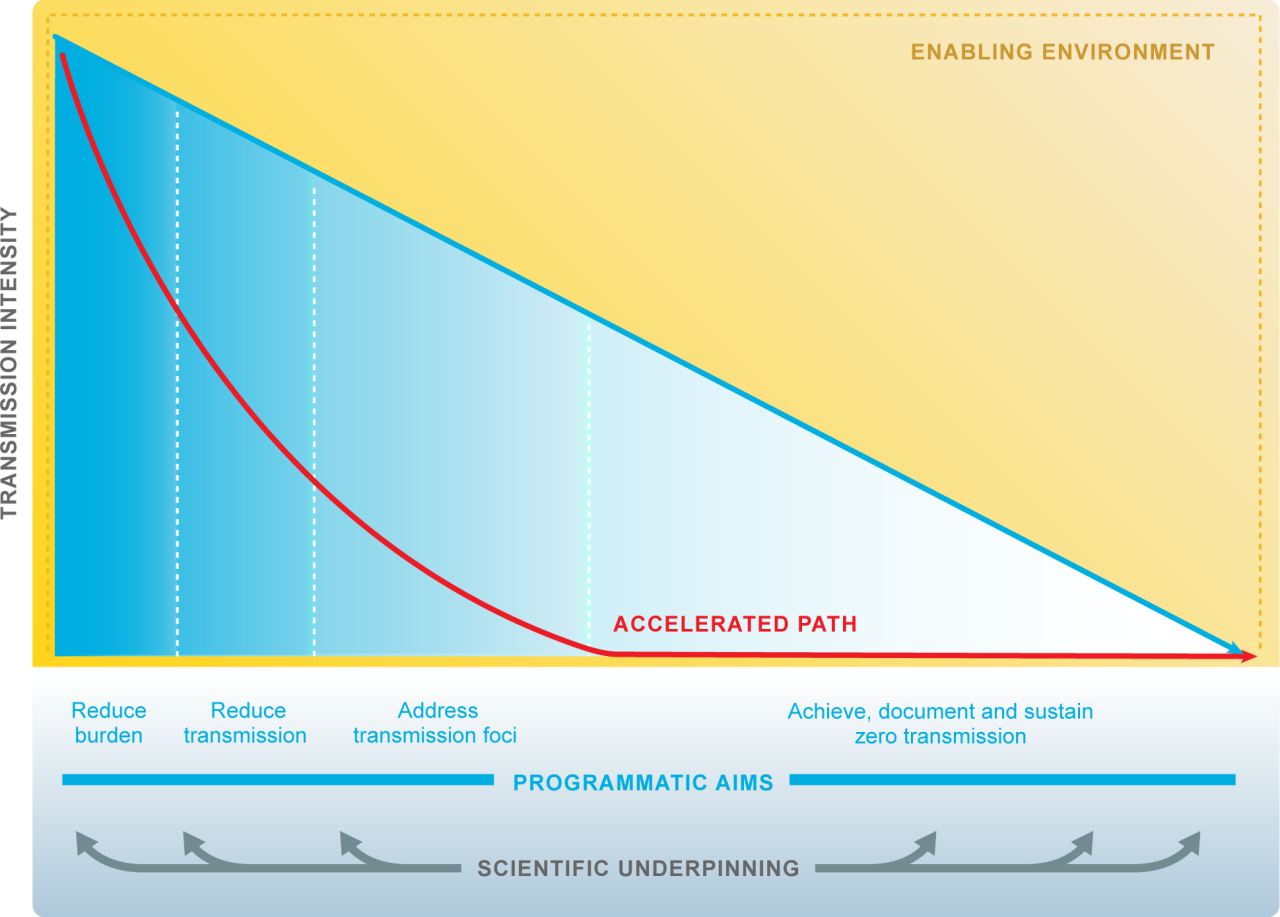
Research accelerates progress towards malaria elimination goals. Research and development responds to the diverse and evolving needs of malaria programmes to underpin elimination and eradication. ‘Scientific underpinning’: research agenda is multidisciplinary and includes all human *Plasmodium* species. Quality evidence informs policies and decision makers. ‘Accelerated path’: technical and operational innovations accelerate progress towards malaria elimination. ‘Enabling environment’: working partnerships between malaria programmes and research institutions. The malaria community and the research community respond effectively to opportunities afforded by other sectors, e.g., urbanisation, education. The malaria community and the research community respond effectively to threats, e.g., natural disasters, conflict, stimulation of career progression and scientific leadership from malaria endemic countries, and commitment to national malaria elimination goals by Ministries of Health, Finance, Science, Education, and Tourism.

Some key points emerge from experiences in elimination countries and are worth clarifying, because they frame the context for evaluation of new tools to accelerate progress. First, elimination has been progressing using current tools and strategies; second, transmission intensity varies widely between and within countries with different mosquitoes and parasite species as well as different health systems and a myriad of varying challenges to the scale-up of interventions; in addition, programmatic goals evolve as transmission changes (Fig 2). The reduction of transmission may progress in a highly variable fashion, affected by ecologic (e.g., climate and outbreaks), biologic (e.g., vector or parasite resistance), and operational (e.g., health delivery system, sociopolitical and -economic status) challenges. Moreover, while some countries have shown durable elimination [23], other countries have come close to but not achieved elimination and then experienced resurgences [24]. New approaches are needed to address vulnerability and receptivity so that elimination can be achieved and sustained in spite of predictable risk of importations.

### malERA Refresh process

The malERA Refresh was undertaken against the background of WHO GTS that was unanimously adopted by the World Health Assembly in 2015 as well as the Roll Back Malaria (RBM) AIM framework [2,3]. Although focussed on malaria, the malERA process itself can be a useful model for defining the research needs, strategies, and portfolios to eliminate and eradicate neglected tropical diseases (NTDs).

**Fig 3:**
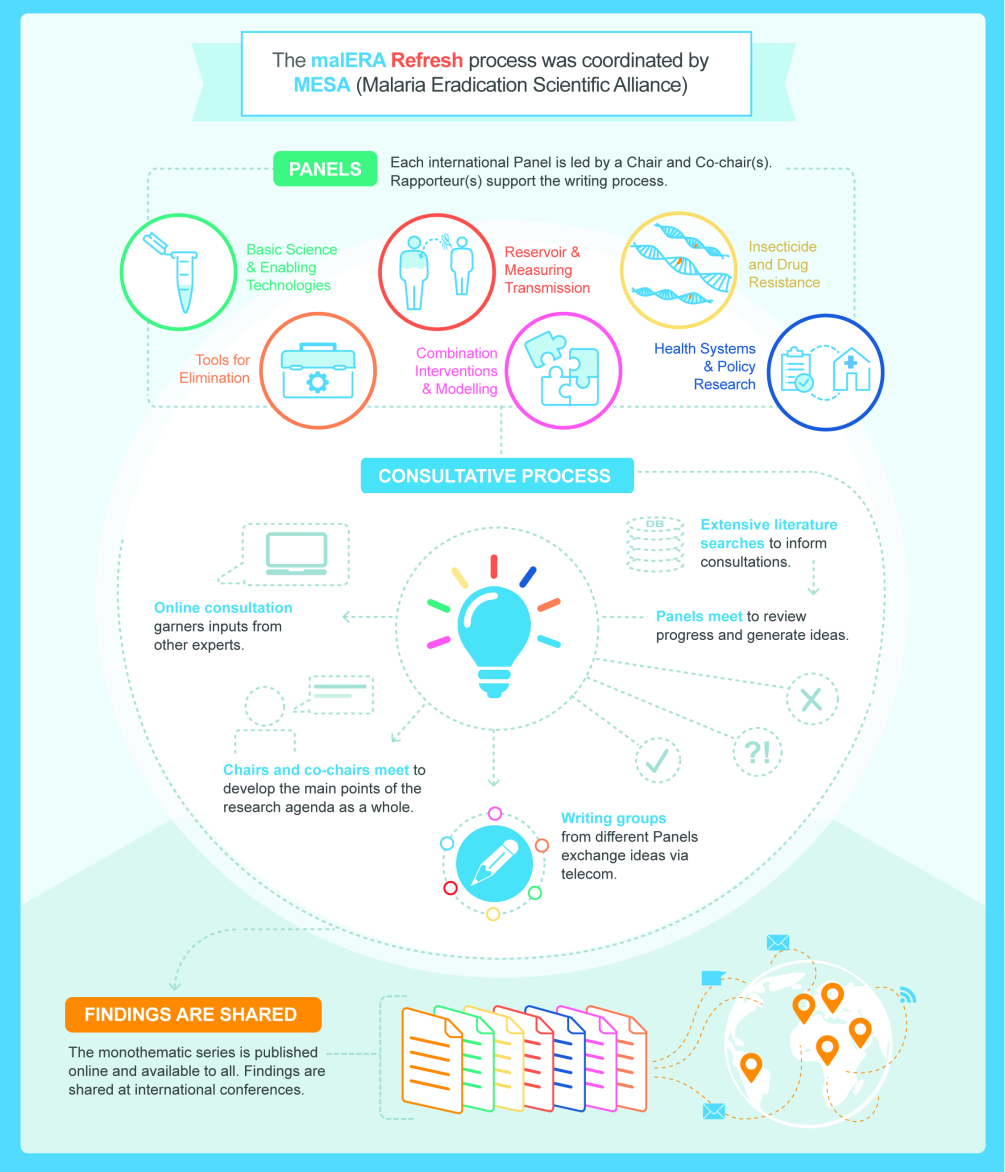
malERA Refresh process. malERA, Malaria Eradication Research Agenda; MESA, Malaria Eradication Scientific Alliance.

The malERA Refresh process was overseen by a leadership group composed of Regina Rabinovich (chair, ISGlobal Barcelona Institute for Global Health and Harvard T.H. Chan School of Public Health), Pedro Alonso (WHO Global Malaria Programme), Marcel Tanner (Swiss TPH), and Dyann Wirth (Harvard T.H. Chan School of Public Health), and each consultative panel was led by a chair and 1 or 2 cochairs [25]. The process was managed by the MESA Secretariat (ISGlobal Barcelona Institute for Global Health). Diverse expert panels of scientists, programme managers, and decision makers were convened for 6 thematic areas. The themes of the panels were adapted from the original malERA, reflecting the evolution of the knowledge base even since the first malERA process in 2011. One panel examined tools for elimination (vector control, vaccines, diagnostics, and drugs), one panel tackled the application of mathematical modelling to the challenges of combining interventions, and the health systems panel also addressed policy research. New panels were created, one to look at the infectious reservoir and one focussed on resistance to antimalarial drugs and insecticides (for the full list of panels, see Table 2). A systematic literature search was performed for each theme to identify papers published between 2010 and 2016. These papers were supplemented with suggestions from panelists and projects in the MESA Track database of active projects. Each panel had 1 in-person meeting to assess the progress since malERA 2011 and discussed whether there had been adequate efforts to address each area. Taking into consideration the major advances that have taken place since the first malERA consultations, the panels highlighted specific challenges and indicated key opportunities to generate knowledge, tools, and strategies for malaria elimination (Box 1). Cross-links between the panels were ensured by cross-panel participation and an online consultation of main findings (Fig 3).

**Table 2:** Using the themes from the first malERA process as a starting point, malERA Refresh was organised by research themes that are relevant for malaria elimination and eradication and reflect current hypotheses and new thinking.

malERA 2011	malERA Refresh 2017
• Introductory paper • Basic science and enabling technologies • Drugs • Vaccines • Vector control • Diagnosis and diagnostics • Monitoring, evaluation, and surveillance • Modelling • Cross-cutting issues for eradication • Lessons for the future • Role of research in viral disease eradication	• Overview and synthesis paper • Basic science and enabling technologies • *Basic science and enabling technologies remained a key theme*. • Insecticide and drug resistance • *Given the evolving threat and search for solutions to resistance*, *a panel was created to address resistance to insecticides and antimalaria drugs in the malaria elimination context*. • Characterising the reservoir and measuring transmission • *To reflect the evolving and nuanced questions around transmission*, *1 panel examined the complexity of the parasite reservoir and the challenges of measuring transmission*. • Diagnostics, drugs, vaccines, and vector control • *A synthetic assessment of product development for malaria was included*. • Combination interventions and modelling • *A panel was created to tackle the power of combining tools and predicting and increasing their impact using mathematical modelling*. • Health systems and policy research • *A panel was dedicated to the operational challenges of malaria elimination in the context of existing health and social systems*.

**Abbreviation:** malERA, Malaria Eradication Research Agenda.

**Box 1. Examples of challenges and opportunities to generate knowledge, tools, and strategies for malaria elimination**

See the papers in this series for the full description of where science has and has not made progress since malERA and the considerations of the main challenges and exciting opportunities going forward.

**Biology**

- There are significant gaps in the knowledge base and ability to tackle the non-*falciparum Plasmodium* species (*P*. *vivax*, *P*. *ovale*, *P*. *malariae*, *P*. *knowlesi*).
- Applying new technologies including CRISPR-Cas9 mediated gene drives, high-throughput screening, metabolomics, and proteomics will help advance malaria biology.

**Tools and deployment strategies**

- Strategies to stop the expanding resistance to pyrethroids, artemisinins, and partner drugs are urgently needed.
- Tools to detect hypnozoites and *P*. *vivax* vaccine candidates remain to be developed.
- Deploying insecticides with novel modes of action.
- Two areas of promise for drug development are applying the controlled human malaria infection (CHMI) models as a bridge to field efficacy of transmission-blocking activity and high-throughput phenotypic screening for the ‘neglected’ product profiles, including hypnozoites and gametocytes.
- Novel approaches to vector control tools are beginning to be explored, including using drugs for vector control.
- Opportunities are emerging regarding monoclonal antibodies for passive immunity.

**Understanding transmission and tackling residual transmission**

- Major questions in understanding transmission remain, from gametocyte biology to characterising and detecting the infectious reservoir.
- Advances are needed in entomological sampling, analysis, and entomological surveillance systems.
- Innovation in genomics, serology, and geospatial tools can help sampling, validating the absence of malaria transmission, and measuring receptivity.

**Malaria programmes and systems**

- Questions remain around the best composition, phasing, and threshold triggers for intervention packages in different settings and as programmes advance along the elimination continuum.
- An area of promise for malaria programmes is testing and validating essential, collectable, and actionable data for programmatic decision-making.
- Advances in molecular technologies will help surveillance of resistance to insecticides and drugs.
- Strategies for deploying future tools in the field need to be tested and modelling can guide testing.
- Opportunities using systems-thinking approaches to identify where in the health system effectiveness of interventions is lost and can be recovered.

A final meeting of all panel leaders reviewed results of this process and identified cross-cutting themes that arose across several panels. These are described further in this paper: surveillance, implementation science, and transmission and persistence. In addition, 2 areas—entomology and *P*. *vivax* malaria—were recognised as research areas that were consistently failing to garner adequate resources and thus scientific engagement. Rather than define specific areas for prioritisation, this research agenda lays out the rationale, context, and relevance for a range of interlinked areas.

### Cross-cutting priority research areas

#### Surveillance and towards surveillance–response approaches

Malaria programmes continuously need data to direct their actions and resources, to gauge their impact, and, particularly in the elimination context, to reorient their tools and strategies to clear infections and stop transmission. The recent Ebola and Zika emergencies have highlighted the critical role of strong health systems with diligent surveillance to enable rapid responses. Surveillance is considered so fundamental to the malaria programme across the transmission spectrum that it represents 1 of the 3 pillars of the GTS [2]. Surveillance itself is an intervention and must be adapted to the respective epidemiological, health, and social system settings [12,26]. Information gleaned from surveillance also informs the rational incorporation of new interventions. In the context of elimination, however, surveillance must be both systematic and sufficiently robust to capture the diminishing number of cases of disease. As elimination nears, surveillance systems must be capable of correctly assessing the infection burden and direct actions; for example, if surveillance data show very few cases, then the programme action can pivot to a reactive approach to treatment around the index patient. Post elimination, surveillance systems must be capable of identifying cases that are reintroduced to prevent resumption of local transmission.

Surveillance platforms like the District Health Information Software 2 (DHIS 2) are being used to collect facility and community data across diseases. When fully functional, such platforms collect dynamic quality-assured information that can be analysed to track temporal and spatial changes in transmission [26,27]. High-quality information systems that collect real-time data from incoming cases can spot early warning signals of drug resistance, reintroduction, and resurgence. High-resolution platforms based on geographic information systems have been developed that collect, integrate, and share relevant data with various audiences [27]. These surveillance–response systems are particularly useful for the detection of and response to unevenly distributed transmission foci with sufficient detail as to depict the single-household or hamlet level and are key to targeting the operational response. In addition to collecting information on malaria infections, a quality malaria surveillance system should assess drug efficacy against the parasites and assess mosquito vector populations and insecticide resistance phenotypes [27,28]. The metrics to best provide this information are still under evaluation.

Research is needed on 2 levels: to better understand low and zero transmission and to develop measures that can be used by programmes. As countries approach elimination, validated epidemiological and entomological markers and efficient sampling strategies will be required to detect transmission at low levels and to confirm the absence of transmission—i.e., the challenge of “measuring zero”. Molecular and serological approaches are being evaluated. For example, identifying and responding to transmission foci would benefit from rapid and noninvasive diagnostic tools that can be applied in nonclinical settings [27]. The balance between predictive value and clinical or public health utility of diagnostic testing will differ in different epidemiologic settings, e.g., as incidence declines, more test-positive cases will be false positives. There are open questions regarding the programmatic impact of new tools to identify subpatent infections that might sustain malaria parasite transmission in some settings [27,29]. The critical balance is that the data collected need to be informative for the programme but also practical in terms of collection and interpretation. The concept of “minimal essential data” describes the balance between a collectable dataset and an informative one, such that programmes can respond to the data [26,28]. As a malaria programme progresses towards elimination, the data requirements will be continually changing and what is deemed “essential” data will also change. There is a need to build an evidence base for effective programmatic responses, e.g., analysis of the systems for data collection, analysis and response to minimise effectiveness decay, developing a portfolio of effective programmatic responses to surveillance data [26], and using modelling and operational research to test specific questions that could facilitate programme performance [12].

#### Implementation science

In contrast to the apparent simplicity of programmes that depend on a single intervention (e.g., vaccines), malaria programmes use a diverse set of tools in an integrated approach to prevent, detect, and treat infections. While the key elements (surveillance, diagnosis, treatment, and prevention) are constant, there are important nuances and evolution for each element as transmission declines. As new tools become available, they need to be integrated into the existing intervention package(s). A critical challenge in malaria elimination is finding the optimal combination of interventions to maximise impact and mitigate the risk of resistance and to modify this package in a timely fashion to respond to the increasingly focal and rapidly changing transmission environment. Interventions have to be introduced, altered, replaced, or possibly withdrawn through adaptive strategies responding to shifting transmission, emerging resistance, and response to unique community issues and needs.

Achieving universal coverage of preventive and curative interventions ‘is one of the biggest opportunities to have a major impact on global mortality and morbidity’ and is also on the critical pathway to elimination [30]. The programmes currently testing MDA approaches are providing evidence of the relevance of community engagement and the need for high uptake of interventions. Health systems and community engagement are both recognised as critical elements in achieving high coverage, but research to define the successful operational criteria is still needed; social science methods have not been fully applied to overcome these challenges [26].

The efficacy of individual interventions is determined through a rigorous set of well-powered comparative trials to answer very specific questions that quantify the potential for impact under controlled circumstances. Under these ideal conditions of very high coverage and adequate use, the efficacy of an intervention equals its effectiveness. Under real field conditions, measurable ‘effectiveness decay’ results from the impact of key elements of the health system, including challenges in financing, procurement, work force, supply chain, and adherence. However, the drivers of effectiveness decay vary and depend on the setting, i.e., unique cultural and/or health systems [26]. malERA 2011 underlined the need to establish a tool for analysing effectiveness decay within a health system, akin to a diagnostic tool for the system itself. It would allow the malaria programme to identify bottlenecks, test different approaches to overcome them, and thus minimise effectiveness decay [31]. Unfortunately, so far, too little investment and progress have been seen in this area and work to understand and mitigate effectiveness decay remains a priority [26].

#### Transmission and persistence

In elimination settings, the malaria programme takes on an added focus: understanding the nuances that contribute to continued transmission in scenarios of low parasitaemia and low incidence and to the parasite’s persistence in host and vector. malERA 2011 stressed the importance of the infection and the transmission reservoir and catalysed a search for tools to identify and interrupt transmission [1,21,22,32,33]. Notably, the concept of a drug combination Single Encounter Radical Cure and Prophylaxis (SERCaP) was developed [21] (see Glossary in Table 1). Today, new chemical entities with a ‘single encounter, radical cure’ profile are undergoing early clinical development. The concept of SERCaP was that it could eliminate all parasites from the human (including the long-lived hypnozoites) in a single encounter suitable for mass administration (including administration to healthy people and the consequent need of a very good safety profile) and prophylaxis for at least 1 month after treatment, to outlast the typical development period of plasmodia parasites in anopheline mosquitoes. Today, new chemical entities with a ‘single encounter, radical cure’ profile are undergoing early clinical development [29]. malERA 2011 expanded the concept of transmission-blocking vaccines to the broader array of VIMT targets (vaccines that interrupt malaria parasite transmission), which can be achieved at several stages of the parasite life cycle, not just the sexual or mosquito stages, as in classical transmission-blocking vaccines [22]. Several VIMT candidates for *P*. *falciparum* are in the development pipeline. Although *P*. *vivax* is now included in the Malaria Vaccine Technology Roadmap strategic goals, VIMTs for *P*. *vivax* have not advanced [29].

Research to characterise the transmission reservoir has evolved to a focus on the role of low-density infections undetected by microscopy or current RDT in transmission [27]. Understanding determinants of the risk of infectiousness, understanding at what level of parasitaemia these are important for sustained transmission, and devising metrics and tools to measure and target transmission are proposed as key needs [26,27,29,34]. Recently, a highly sensitive RDT has been launched and demonstration studies are being planned to test how and when to use this new tool [35].

Measuring zero transmission is a requisite for programmes that seek to eliminate malaria and for evaluating tools in the development pipeline that aim to interrupt or reduce transmission. Validated, measurable epidemiological and entomological indicators of transmission are needed. The papers in this series discuss the research agenda and potential solutions [12,26–29,34].

Transmission needs to be reliably measured both at the mosquito and human levels, but the tools available today only provide proxies for true transmission. Currently, vector control tools are not able to interrupt all malaria transmission, and ‘residual transmission’ can persist even in areas with good vector control coverage (see Glossary in Table 1). Residual transmission is now recognised as a target for investigation and intervention, but there is no consensus yet on how to quantify this concept. Novel tools to interrupt residual transmission as a complement to traditional vector control are under development and include toxic sugar-baited traps, endectocides, and targeted larviciding [27,29].

Gametocyes are the transmissible form of the parasite from humans and present a biological opportunity because they are relatively few in number compared to other parasite stages. Drug candidates with gametocytocidal properties are early in the pipeline and will need to be tested for their ability to arrest the transmission cycle, and the search for tractable vaccine targets that attack gametocytes in the human host needs to continue [29,34]. Knowledge of the drivers controlling gametocyte production is poor, e.g., understanding what environmental conditions might favour an increased production of gametocytes and facilitate transmission [27]. Moreover, there is a need to better define the relationship between gametocyte densities and transmission for both *P*. *falciparum* and *P*. *vivax*. Reliable biomarkers for both gametocytes and hypnozoites would enable this.

The key determinants for persistence and recrudescence remain to be established. In highly seasonal settings, it has been demonstrated that humans can act as the parasite reservoir by carrying gametocytes at levels beneath detection of current diagnostics, but the role of the mosquito as a reservoir during those months is still poorly understood [27,34].

### Major neglected areas critical to elimination

#### Entomology

Despite the indisputable merit of vector control tools in the reductions of malaria morbidity and mortality and increasing vector resistance against insecticides, investment in this area has lagged [36]. This scenario extends from basic research through product development and training.

Currently, collecting entomological data is laborious and trained entomologists and staff are scarce. Programmes such as TDR and the US President's Malaria Initiative have recognised the need for improving national capacities for entomological monitoring and support training efforts in some countries [37,38]. The recently adopted 'Global Vector Control Response’ report marks a significant commitment of WHO and member states to strengthen vector control within a collaborative framework [39]. Recent global outbreaks of other vector-borne diseases such as Zika and chikungunya highlight the need for countries to garner the necessary support for strengthening capacity in entomology and vector control that is also relevant for malaria. malERA Refresh panelists agreed that medical entomology must have a central role in the global health curriculum and in the training curriculum for Ministry of Health staff.

The efficacy of available vector control tools is diminished by residual transmission and the enormous behavioural plasticity and biological variability of malaria vectors and is threatened by the capacity of the mosquito to develop resistance in the face of high pressure from interventions. The papers in this malERA Refresh series offer potential solutions to be developed and tested [27–29,34].

Novel entomological markers for transmission are needed because the traditional measure—entomological inoculation rate (EIR)—is not a practical or easily reproducible metric in lower-transmission settings [27]. The gap in data collection capacities needs to be addressed by testing and validating what constitute minimal essential, collectable, and actionable data. New technologies are needed to generate robust data on species distribution, temporal and spatial biting patterns, and spread of insecticide resistance, which would be actionable data from entomologic surveillance in the future [27,29].

#### Vivax malaria (and 3 other species)

Five species of *Plasmodium* infect humans. *P*. *falciparum* has been a global priority due to its role as a driver of mortality and severe disease. However, *P*. *vivax* is geographically the most widely distributed form of human malaria, causes 13.8 million cases every year, and is associated with both significant morbidity and a risk for mortality [9]. The research agenda presented in the malERA Refresh series is relevant to *P*. *falciparum* and *P*. *vivax*; specific challenges posed by *P*. *vivax* are highlighted in the thematic papers and here.

There are important differences in the biology of *P*. *vivax*, particularly its ability to remain quiescent in the liver, different kinetics and appearance of infectious gametocytes, and significant differences in its clinical presentation and risk of recurrence. Unique drugs, diagnostics, and different targets for vaccine development and strategies are required beyond what is available today.

malERA 2011 acknowledged hypnozoites as a challenge to *P*. *vivax* elimination, and this remains the case, with a lack of diagnostics to identify carriers and safe efficacious treatments to clear them [1,29]. Proteomic and metabolomic techniques have been suggested as possible research tools to detect hypnozoites; additional in vitro studies are needed to expand current knowledge of their biology and metabolism [34].

Countries with *P*. *falciparum* and *P*. *vivax* malaria seek to eliminate the disease entirely rather than a single species. Thus, tackling *P*. *vivax* was considered critical in malERA 2011 and, while the biological and epidemiological knowledge base has significantly improved, there is still a relatively weak pipeline of drugs and vaccines [1,27,29,34].

Tafenoquine is in late-stage development. It is a candidate drug that results in radical cure of all circulating parasites and *P*. *vivax* hypnozoites in a single treatment and confers prophylaxis for several weeks posttreatment. Results from a Phase III clinical trial show that single-dose tafenoquine reduces risk of relapse in patients with *P*. *vivax* malaria [29,40]. When tafenoquine becomes available, it will not remove the need to test for glucose-6-phosphate dehydrogenase (G6PD) deficiency, which affects 350 million people at risk for malaria and remains a considerable obstacle to effective treatment [41]. Novel point-of-care diagnostic tests for G6PD deficiency are currently in late-stage development [29]. In the future, newly developed humanised mouse models could help predict the haemolytic potential of drugs in the pipeline [29,34].

*P*. *knowlesi* poses unique challenges among the 5 malaria species, owing to its zoonotic transmission. WHO convened an Evidence Review Group (ERG) to review existing data on *P*. *knowlesi*, including an upward trend in incidence documented in Malaysia, and identify knowledge gaps. The ERG articulated the need for evidence to better understand the likelihood of human to human transmission [11].

### Looking forward

Innovation and problem solving tailored to the local setting are critical to the long-term success of the global malaria programme. Three types of innovation need to be pursued: iterative, breakthrough, and integrated. malERA Refresh is replete with examples: drugs to overcome resistance, gene drive as a transformative technology, and the acceleration hypothesis as a testable approach to elimination and its interaction with the health system in highly endemic countries. To pursue the opportunities proposed here for accelerating elimination, a diverse landscape of funders is needed to prioritise research objectives according to their strategic plans and stakeholders’ needs. A diligent monitoring of the uptake of the research questions in this agenda and the impact of the evolving evidence base will be essential to keep the malaria community on course.

## Supporting information

S1 Translation Spanish translation of abstract. (DOCX)

S2 Translation French translation of abstract. (DOCX)

## Abbreviations

AIM: Action and Investment to defeat Malaria 2016–2030
CHMI: controlled human malaria infection
CRISPR: clustered regularly interspaced short palindromic repeats
DHIS 2: District Health Information Software 2
EIR: entomological inoculation rate
ERG: Evidence Review Group
G6PD: glucose-6-phosphate dehydrogenase
GTS: Global Technical Strategy for Malaria 2016–2030
malERA: Malaria Eradication Research Agenda
MDA: mass drug administration
MESA: Malaria Eradication Scientific Alliance
NTD: neglected tropical disease
RBM: Roll Back Malaria Partnership
RDT: rapid diagnostic test
SAG: Strategic Advisory Group
SERCaP: Single Encounter Radical Cure and Prophylaxis
VIMT: vaccines that interrupt malaria parasite transmission
WHO: World Health Organization

## References

[1] Alonso PL, Brown G, Arevalo-Herrera M, Binka F, Chitnis C, Collins F, et al A research agenda to underpin malaria eradication. PLoS Med. 2011;8(1):e1000406 doi: 10.1371/journal.pmed.1000406 21311579 10.1371/journal.pmed.1000406 PMC3026687

[2] World Health Organization. Global technical strategy for malaria 2016–2030. Geneva: WHO; 2015. Contract No.: 17 March.

[3] Roll Back Malaria Partnership. Action and investment to defeat malaria 2016–2030: for a malaria-free world Geneva: WHO; 2015 Available from:http://rollbackmalaria.com/. Date accessed 2017 Nov 1.

[4] End malaria 2040. From Aspiration to Action: What Will It Take to End Malaria?; 2016.

[5] World Health Organization. WHO Strategic advisory group (SAG) on Malaria. Eradication of malaria, Report by the Secretariat. Geneva; 2017 18.05.2017.

[6] World Health Organization. A framework for malaria elimination. Geneva; 2017. Contract No.: ISBN: 978 92 4 151198 8.

[7] Melinda French Gates. BMGF Malaria Forum Keynote Address: Bill & Melinda Gates Foundation; 2007 [Prepared remarks by Melinda French Gates, co-chair BMGF]. Available from: https://www.gatesfoundation.org/media-center/speeches/2007/10/melinda-french-gates-malaria-forum. Date accessed 2017 Oct 10.

[8] Roberts L, Enserink M. Malaria. Did they really say… eradication? Science. 2007;318(5856):1544–5. doi: 10.1126/science.318.5856.1544 18063766 10.1126/science.318.5856.1544

[9] World Health Organization. World Malaria Report. Geneva; 2016 13.12.2017. Report No.: ISBN: 978 92 4 151171 1.

[10] World Health Organization. Eliminating malaria. Geneva: WHO; 2016, 20 May.

[11] World Health Organization. Malaria Policy Advisory Committee meeting report Geneva; 2017, March. Report No.: WHO/HTM/GMP/MPAC/2017.8.

[12] malERA Refresh Consultative Panel on Combination Interventions and Modelling. malERA: An updated research agenda on combination interventions and modelling for malaria elimination and eradication. PLoS Med. 2017; 14(11): e1002453 doi: 10.1371.journal.pmed.1002453 10.1371/journal.pmed.1002453 PMC5708628 29190295

[13] MESA Track database [Internet]. [cited 2017]. Available from: http://www.malariaeradication.org/mesa-track. Date accessed 2017 Oct 10.

[14] World Health Organization. WHO malaria terminology. Geneva: WHO; 2016. Contract No.: 1 October.

[15] Epstein JE. Taking a bite out of malaria: controlled human malaria infection by needle and syringe. Am J Trop Med Hyg. 2013;88(1):3–4. doi: 10.4269/ajtmh.2013.12-0715 23303797 10.4269/ajtmh.2013.12-0715 PMC3541742

[16] Mordmuller B, Supan C, Sim KL, Gomez-Perez GP, Ospina Salazar CL, Held J, et al Direct venous inoculation of *Plasmodium falciparum* sporozoites for controlled human malaria infection: a dose-finding trial in two centres. Malar J. 2015;14:117 doi: 10.1186/s12936-015-0628-0 25889522 10.1186/s12936-015-0628-0 PMC4371633

[17] Gomez-Perez GP, Legarda A, Munoz J, Sim BK, Ballester MR, Dobano C, et al Controlled human malaria infection by intramuscular and direct venous inoculation of cryopreserved *Plasmodium falciparum* sporozoites in malaria-naive volunteers: effect of injection volume and dose on infectivity rates. Malar J. 2015;14:306 doi: 10.1186/s12936-015-0817-x 26245196 10.1186/s12936-015-0817-x PMC4527105

[18] Dan Strickman. Definition adapted from personal communication. 2017.

[19] WHO Alliance for Health Policy and System Research. Systems thinking for health systems strengthening. 2009.

[20] Zachariah R, Harries AD, Ishikawa N, Rieder HL, Bissell K, Laserson K, et al Operational research in low-income countries: what, why, and how? Lancet Infect Dis. 2009;9(11):711–7. doi: 10.1016/S1473-3099(09)70229-4 19850229 10.1016/S1473-3099(09)70229-4

[21] malERA Consultative Group on Drugs. A research agenda for malaria eradication: drugs. PLoS Med. 2011;8(1):e1000402 doi: 10.1371/journal.pmed.1000402 21311580 10.1371/journal.pmed.1000402 PMC3026688

[22] malERA Consultative Group on Vaccines. A research agenda for malaria eradication: vaccines. PLoS Med. 2011;8(1):e1000398 doi: 10.1371/journal.pmed.1000398 21311586 10.1371/journal.pmed.1000398 PMC3026701

[23] Smith DL, Cohen JM, Chiyaka C, Johnston G, Gething PW, Gosling R, et al A sticky situation: the unexpected stability of malaria elimination. Philosophical transactions of the Royal Society of London Series B, Biological sciences. 2013;368(1623):20120145 doi: 10.1098/rstb.2012.0145 23798693 10.1098/rstb.2012.0145 PMC3720043

[24] Cohen JM, Smith DL, Cotter C, Ward A, Yamey G, Sabot OJ, et al Malaria resurgence: a systematic review and assessment of its causes. Malar J. 2012;11:122 doi: 10.1186/1475-2875-11-122 22531245 10.1186/1475-2875-11-122 PMC3458906

[25] Malaria Eradication Scientific Alliance. MESA malERA Refresh [updated 2017. Available from: http://www.malariaeradication.org/. Date accessed 2017 Oct 10.

[26] malERA Refresh Consultative Panel on Health Systems and Policy Research. malERA: An updated research agenda for health systems and policy research in malaria elimination and eradication. PLoS Med. 2017; 14(11): e1002454 doi: 10.1371/journal.pmed.1002454 10.1371/journal.pmed.1002454 PMC5708613 29190289

[27] malERA Refresh Consultative Panel on Characterising the Reservoir and Measuring Transmission. malERA: An updated research agenda for characterising the reservoir and measuring transmission in malaria elimination and eradication. PLoS Med. 2017; 14(11): e1002452 doi: 10.1371/journal.pmed.1002452 10.1371/journal.pmed.1002452 PMC5708619 29190279

[28] malERA Refresh Consultative Panel on Insecticide and Drug Resistance. malERA: An updated research agenda for insecticide and drug resistance in malaria elimination and eradication. PLoS Med. 2017; 14(11): e1002450 doi: 10.1371/journal.pmed.1002450 10.1371/journal.pmed.1002450 PMC5708661 29190671

[29] malERA Refresh Consultative Panel on Tools for Elimination. malERA: An updated research agenda for diagnostics, drugs, vaccines and vector control in malaria elimination and eradication. PLoS Med. 2017; 14(11): e1002455 doi: 10.1371/journal.pmed.1002455 10.1371/journal.pmed.1002455 PMC5708606 29190291

[30] Roll Back Malaria Partnership. Global Malaria Action Plan (GMAP) for a malaria-free world. Geneva; 2008.

[31] malERA Consultative Group on Health Systems and Operational Research. A research agenda for malaria eradication: health systems and operational research. PLoS Med. 2011;8(1):e1000397 doi: 10.1371/journal.pmed.1000397 21311588 10.1371/journal.pmed.1000397 PMC3026705

[32] malERA Consultative Group on Diagnoses and Diagnostics. A research agenda for malaria eradication: diagnoses and diagnostics. PLoS Med. 2011;8(1):e1000396 doi: 10.1371/journal.pmed.1000396 21311583 10.1371/journal.pmed.1000396 PMC3026696

[33] malERA Consultative Group on Vector Control. A research agenda for malaria eradication: vector control. PLoS Med. 2011;8(1):e1000401 doi: 10.1371/journal.pmed.1000401 21311587 10.1371/journal.pmed.1000401 PMC3026704

[34] malERA Refresh Consultative Panel on Basic Science and Enabling Technologies. malERA: An updated research agenda on basic science and enabling technologies in malaria elimination and eradication. PLoS Med. 2017; 14(11): e1002451 doi: 10.1371/journal.pmed.1002451 10.1371/journal.pmed.1002451 PMC5708601 29190277

[35] Alere Launches the Alere Malaria Ag P.f, the First-Ever Rapid Test to Screen Malaria Infection in Asymptomatic Individuals [press release]. Alere Inc., 25.04.2017 2017.

[36] Bhatt S, Weiss DJ, Cameron E, Bisanzio D, Mappin B, Dalrymple U, et al The effect of malaria control on *Plasmodium falciparum* in Africa between 2000 and 2015. Nature. 2015;526(7572):207–11. doi: 10.1038/nature15535 26375008 10.1038/nature15535 PMC4820050

[37] President’s Malaria Initiative. Entomological Monitoring [Tools and training]. Available from: https://www.pmi.gov/how-we-work/technical-areas/entomological-monitoring. Date accessed 2017 Oct 10.

[38] WHO TDR the Special Programme for Research and Training in Tropical Diseases. Capacity strengthening [Available from: http://www.who.int/tdr/capacity/en/. Date accessed 2017 Oct 10.

[39] World Health Organization. Global vector control response 2017–2030. Geneva; 2017, May 31.

[40] GSK and MMV announce positive headline phase III results showing single-dose tafenoquine reduces risk of relapse in patients with Plasmodium vivax malaria. [press release]. 12.06.2017 2017.

[41] Thriemer K, Ley B, Bobogare A, Dysoley L, Alam MS, Pasaribu AP, et al Challenges for achieving safe and effective radical cure of Plasmodium vivax: a round table discussion of the APMEN Vivax Working Group. Malar J. 2017;16(1):141 doi: 10.1186/s12936-017-1784-1 28381261 10.1186/s12936-017-1784-1 PMC5382417

