# Supplementary material for: malERA: An updated research agenda for malaria elimination and eradication
Source: PLoS Med. 2017 Nov 30;14(11):e1002456. doi: 10.1371/journal.pmed.1002456 (PMC5708604; doi:10.1371/journal.pmed.1002456)
Supplement: S1 Translation — (DOCX) [file pmed.1002456.s001.docx]

L’objectif d'un monde exempt de paludisme présente des défis scientifiques passionnants ainsi que des avantages incomparables pour la santé, l'équité et l'économie. L'OMS et différents pays sont en train d’établir des objectifs ambitieux pour réduire la charge mondiale puis éliminer le paludisme par le biais de la «Stratégie technique mondiale» et par son élimination d'ici 2020 dans 21 pays. On ne peut que féliciter les efforts pour atteindre ces cibles. Cependant, le besoin d'innovation afin de parvenir à ces objectifs, de soutenir l’élimination et d’accélérer la fin du paludisme est plus important que jamais. Plus de 180 experts en diverses disciplines se sont engagés dans le processus d’actualisation du programme de recherche sur l'éradication du paludisme (MalERA). Il en résulte un programme de recherche et de développement visant à accélérer l'élimination du paludisme et, à plus long terme, à transformer notre capacité à rendre possible l'éradication.
