# Supplementary material for: malERA: An updated research agenda for malaria elimination and eradication
Source: PLoS Med. 2017 Nov 30;14(11):e1002456. doi: 10.1371/journal.pmed.1002456 (PMC5708604; doi:10.1371/journal.pmed.1002456)
Supplement: S2 Translation — (DOCX) [file pmed.1002456.s002.docx]

Lograr un mundo libre de malaria conlleva, además de emocionantes retos científicos,  abrumadores beneficios tanto en términos de salud y equidad como en términos económicos. La Organización Mundial de la Salud (OMS) y los países están definiendo ambiciosas metas para reducir la carga y eliminar la malaria a través de la “Estrategia Técnica Mundial” y 21 países aspiran a eliminar la enfermedad en 2020. El compromiso para lograr estos objetivos debería ser celebrado. No obstante, la necesidad de innovación para poder alcanzar estas metas, mantener la eliminación y acelerar el avance hacia un mundo libre de malaria es mayor que nunca. Más de 180 expertos de múltiples disciplinas colaboraron en el proceso de actualización de la agenda de investigación para la erradicación de la malaria (malERA), con el objetivo de abordar problemas que necesitan una solución.  El resultado es una agenda de investigación y desarrollo para acelerar la eliminación de la malaria, y a largo plazo, transformar nuestras habilidades para que la erradicación sea factible.
